# Evaluation of Microcirculation, Cytokine Profile, and Local Antioxidant Protection Indices in Periodontal Health, and Stage II, Stage III Periodontitis

**DOI:** 10.3390/jcm10061262

**Published:** 2021-03-18

**Authors:** Artem Eldzharov, Dzerassa Kabaloeva, Dmitry Nemeryuk, Aida Goncharenko, Adelina Gatsalova, Elena Ivanova, Igor Kostritskiy, Florence Carrouel, Denis Bourgeois

**Affiliations:** 1 Federal State Budgetary Educational Institution of Higher Education, “North-Ossetian State Medical Academy” of the Ministry of Healthcare of the Russion Federation, Pushkinskaya Str. 40, 362019 Vladikavkaz, Russia; dzera_kabaloeva@mail.ru; 2 Federal State Budgetary Educational Institution of Higher Education, “A.I. Yevdokimov Moscow State University of Medicine and Dentistry” of the Ministry of Healthcare of the Russion Federation, St. Delegatskaya, 20, Building 1, 127473 Moscow, Russia; tatnem82@mail.ru (D.N.); adgoncharenko@mail.ru (A.G.); 3 Clinical Center of Maxillofacial, Plastic Surgery and Dentistry, st. Vuchetich, 9a, 127206 Moscow, Russia; adelina-2507@mail.ru; 4 National Medical Research Center of Rehabilitation and Balneology of the Ministry of Healthcare of the Russian Federation, St. Novy Arbat, 32, 121099 Moscow, Russia; epivan7@gmail.com; 5 Federal State Budgetary Educational Institution of Higher Education, Irkutsk State Medical University of the Ministry of Healthcare of the Russion Federation, Krasnogo Vosstaniya Street, 1, 664003 Irkutsk, Russia; kostrit@mail.ru; 6 Laboratory “Health Systemic Process”, University Lyon 1, EA4129, 69008 Lyon, France; florence.carrouel@univ-lyon1.fr (F.C.); denis.bourgeois@univ-lyon1.fr (D.B.)

**Keywords:** periodontitis, microcirculation, cytokines, antioxidant, active oxygen species, microbiome

## Abstract

Periodontitis, initiated by the subgingival biofilm and modified by the individual’s inflammatory/immune response, has been associated with vascular dysfunction. To analyze microcirculation indices in periodontal tissues and determine the activity of the enzymatic component of antioxidant defense and humoral immunity factors, a single-blind non-invasive clinical trial was realized. Forty subjects, aged from 30 to 65 years, with moderate to severe chronic periodontitis (chronic generalized periodontitis, CGP) vs. 40 subjects as periodontally healthy were recruited. Information such as capillary diameter, capillary blood flow velocity, concentration of pro- and anti-inflammatory cytokines in serum, vascular endothelial growth factor, and enzymatic component of antioxidant protection were taken. The revealed microcirculatory dysfunctions in patients with CGP clearly demonstrate the progressive disorder of periodontal tissue perfusion and oxygenation, the presence of increased vascular permeability and functional failure of the microvascular system in the lesion. Cytokine profile of CGP patients’ blood serum demonstrated a significant increase of interleukin (IL)-1β, IL-6, tumor necrosis factor α (TNF-α), IL-4 levels as well as statistically significant decrease of IL-1ra, IL-10 concentration. Participants with CGP demonstrated a dominant superiority of IgM and IgG levels. In conclusion, these results contribute to a better understanding of potential correlation between microvascular changes and local and systemic markers of inflammation.

## 1. Introduction

Periodontitis is an infectious-inflammatory disease, which is initiated by the subgingival biofilm and modified by the individual’s inflammatory/immune response [1,2]. Resulting from loss of balance between the commensal microbiome and the host response, the pathogenesis of periodontal diseases is mediated by the inflammatory response to bacteria in the dental biofilm [3]. During gingival inflammation, accumulation of infiltrates within the connective tissue led to collagen degradation or fibrotic reaction by stimulating the effects of inflammatory mediators of the connective tissue [4,5,6,7]. In addition, inflammation is associated with pathological angiogenesis and a high number of newly formed blood vessels that can be quantified as micro-vessel density (MVD) [8]. Angiogenesis together with inflammatory infiltrate are associated with the evolution of gingival inflammatory processes [9].

Chronic periodontitis, one of the most common types of periodontitis, has been associated with numerous conditions, including vascular dysfunction [10]. Periodontitis is accompanied by the proliferation of small blood vessels in the gingival lamina propria. Specialized postcapillary venules, termed periodontal high endothelial-like venules, are also present, and demonstrate morphological and functional traits similar to those of high endothelial venules (HEVs) in lymphatic organs [11].

Numerous researches have indicated that microbial contamination plays a dominant role in the formation of periodontitis [12,13,14,15,16,17]. Dispersion of foreign invaders into periodontal complex structures, production of histotoxic enzymes, and prolonged persistence result in progressive microcirculation disorder, increase of free-radical processes, and structural disorganization of cell membranes [18,19]. Similar destructive processes can initiate a complex cascade of immunopathological reactions, often manifested in auto-aggression by the immune system [20,21,22]. As a result, the immune response incidentally involves the host’s cells [23,24].

In the circulatory system, the microvasculature is the link between arterial and venous vessels and depends on a large number of factors acting at the tissue level [25]. The study of the vascular system of the periodontium is important since the microvascular bed is the terminal site where the transport function of the cardiovascular system is realized and transcapillary metabolism is provided, which determines periodontal tissue metabolism. One of the first signs of microcirculation disorder is a local spasm of the bringing arteriolar vessels, as a result of which the intensity of blood flow in the exchange link of the capillary bed decreases. Disorders of transcapillary mass transfer and metabolic processes ultimately lead to increasing tissue hypoxia and ischemia [26].

In addition, a number of specific rheological intravascular effects occur in the microvasculature, such as intravascular aggregation of erythrocytes, temporary blockage of the mouth sections of microvessels with leukocytes, or the appearance of plasma capillaries. These processes are characteristic only for microvessels. This research will address the pathways of inflammation in periodontal diseases by focusing on immunologic mechanisms to elucidate sites of regulation.

The aim of this study was to compare the microcirculation in periodontal tissues of chronic generalized periodontitis (CGP) subjects and healthy periodontal subjects in order to create a comprehensive and innovative treatment framework aimed at correcting the detected disorders. For that, the indices of microcirculation in periodontal tissues, the activity of antioxidant enzymes as well as the expression of molecules of the humoral immune response were analyzed.

## 2. Experimental Section

### 2.1. Study Design and Ethical Approval

This study was designed as a single-blind clinical trial, conducted at the Dental Polyclinic of the North Ossetian State Medical Academy (NOSMA), Russia. The study protocol was reviewed and approved by the institutional Ethics Council of the North Ossetian State Medical Academy (NOSMA) and conducted in accordance with the principles of the Helsinki Declaration of 1975, as revised in 2008. Written informed consent was obtained from all patients.

### 2.2. Samples Collection and Processing

The study comprised 80 patients, aged 30 to 65 years, from a pool consulting to the Dental Polyclinic of the North Ossetian State Medical Academy, Russia between March 2018 and March 2020. Stratification by gender occurred at the enrolment step. Forty patients with CGP were recruited consecutively during the period. Meanwhile, 40 systemically subjects as periodontally healthy were also recruited and assigned to the control group for a comparative study.

Inclusion criteria were patients: age 30 to 65 years old; no systemic illnesses or disorders; not pregnant or breastfeeding; no medical treatment that may impair healing (immunodepression, immunosuppression, diabetes, etc.); more than 20 natural teeth excluding the third molars; ability and willingness to give written informed consent; written agreement to participate in the trial.

Exclusion criteria included tobacco, use of antibiotics or anti-inflammatory drugs in the previous one month, use of medications, such as anti-platelet or anti-coagulant agents, any other concomitant systemic disorder, cardiovascular and diabetes diseases, allergic, infectious diseases and medications affecting periodontal inflammation, patients with inflammatory periodontal diseases requiring immediate and independent medical attention and, any condition requiring premedication before dental treatment, less than 20 natural teeth, no informed consent. Patients with previous periodontal treatment or who were undergoing a course of dental or orthodontic treatment were also excluded.

### 2.3. Classification of Subjects

#### 2.3.1. Classification of Subjects as Periodontally Healthy

The diagnosis of periodontally healthy was made according to the American Academy of Periodontology [27], with some modifications [28]. The comparative group was composed of individuals with clinically healthy periodontal tissues (probing depth (PD) ≤ 3 mm, clinical attachment level (CAL) < 3 mm and ≤10% of sites with bleeding on probing (BOP) after 30 s).

#### 2.3.2. Classification of Subjects with Stage II, III Periodontitis

The diagnosis of moderate to severe chronic periodontitis was assigned to subjects presenting periodontal lesions of stage II, III (i.e., PD ≥ 4 mm, and/or CAL ≥ 4 mm), generalized (>30% of sites), and radiographic evidence of a vertical bone defect of at least 4 mm [28].

### 2.4. Clinical Record

The information through anamnesis included demographic characteristics (smoking, age and gender), medical information (medical history, diabetes, hypertension, heart disease), and dental information (dental history, clinical and radiographic examinations) were collected during a primary session. No further oral-hygiene instructions were provided.

Standardized periodontal monitoring was performed during a second clinical session, one week later. Screening of the clinical assessments were evaluated: PD, CAL using a periodontal probe with a diameter of 0.5 mm (PCV12, Hu-Friedy, Chicago, IL, USA). The final clinical diagnosis was rendered by two experienced periodontist observers. The intra- and inter-examiner coefficients for CAL ranged between 0.78 and 0.85, and between 0.75 and 0.85 for PD.

The level of oral hygiene and the severity of gingival inflammation were respectively determined using the Simplified Oral Hygiene Index (OHI-S) [29] and the BOP [30].

### 2.5. Capillaroscopy

The study of microcirculation and structural features of the gingival tissue capillary network was carried out in the area of the transitional fold of the lower jaw. Computer capillaroscopy method (KK technology, Honiton, UK) using a capillaroscope KK4-01 (“TSAV” JSC Center “Analiz veschestv”, Russia) (Supplementary File 1) was applied to examine with a 300-fold magnification and a resolution of 1.0 μm. The protocol was modified from one of Dababneh et al. (2014) [31]. Subjects was instructed not to eat or drink for 12 h before testing. After 20 min of acclimatization to a constant temperature of 20–22 °C, videos were acquired with subjects in a sitting position (Supplementary File 1). The areas where the microvasculature network formed loops were avoided. At the preliminary stage, the state of the vascular pattern was assessed in order to establish the features of the microvascular system in patients with CGP. Quantitative indicators were registered: (i) the capillary dimensions (μm) that correspond to the length of the visible part of the capillary and the ratio of the diameters of the sections (arterial, transitional, and venous); (ii) the linear blood flow velocity in isolation in the arterial and venous sections (μm/s) that is the speed of movement of the blood cells, plasma in the capillary bed. The travel speed should be optimal, i.e., effectively ensure metabolism: in tissue—substances for tissue nutrition; from fabric—metabolic products; and (iii) the volumetric velocity of capillary blood flow in the arterial and venous sections (μm^3^/s) sets the flow rate of capillary blood through the section of the capillary bed in sections. Information about this indicator makes it possible to quantify the state of transcapillary metabolism and identify the type of exchange.

The analysis of microcirculatory parameters was carried out in two modes—manual and automatic. In the manual mode, the linear indicators of the capillary network were determined by calibrating the pixel distance of the obtained images with the same distance on the micro-ruler. The automatic mode of measuring the parameters was carried out using the C-Scope 0.90a software. During capillaroscopy, all patients were assessed qualitative (capillary shape, its deviations from the classical shape, tortuosity, etc.) and quantitative morphological characteristics of capillaries: the diameter of the arterial and venous parts of the capillaries. The diameter of each of the capillary sections (and was determined by measurement. It should be noted that the studied values of the microvasculature, obtained in manual and automatic measurement modes, did not differ significantly (*p* > 0.05).

### 2.6. Sampling of Fluids

#### 2.6.1. Sampling of Blood

Venous blood samples were collected under standard aseptic condition from the cubital vein, on an empty stomach in the morning.

#### 2.6.2. Sampling of Saliva

Subjects were instructed not to consume food or beverages except water for 2 h before saliva collection, which was done in the morning between 10:00 a.m. and 12:00 p.m. Each subject was asked to collect 2 mL of saliva by expectoration in polypropylene tubes (Eppendorf, Hamburg, Germany). Immediately after collection, the saliva sample was centrifuged at 3000 rpm for 10 min at 4 °C to remove bacteria, exfoliated epithelial cells, and debris. The supernatant was taken and used as an analyte for enzyme immunoassay.

### 2.7. Determination of VEGF and Immunoglobulin Levels in the Saliva

Determination of concentration of vascular endothelial growth factor (VEGF), secretory immunoglobulin A (sIgA), IgM, and IgG in saliva was carried out by enzyme-linked immuno-sorbent assay (ELISA) from Vector Best (Russia). Streptavidin-horseradish peroxidase (HRP) conjugated with specific monoclonal mouse antibodies against human VEGF, sIgA, IgM and IgG, and tetramethylbenzidine (TMB) as substrate was used according to the standard manufacturer’s protocol (Vector Best). The optical density was measured at 450 nm. The concentrations were determined using calibration curves obtained according to standard manufacturer’s protocols.

### 2.8. Determination of Cytokine Levels in the Serum

Determination of concentration of pro- and anti-inflammatory cytokines (interleukin (IL)-1β, IL-6, tumor necrosis factor α (TNF-α), IL-4, IL-1ra, IL-10) in serum was carried out by using commercial ELISA kits (Cytokine, Moscow, Russia) according to the manufacturer’s instructions. The reaction was developed using streptavidin-HRP with the TMB, and the optical density was measured at 450 nm. The concentrations were determined using calibration curves obtained according to standard manufacturer protocols.

### 2.9. Determination of the Antioxidant Enzymes Activity

The state of the enzymatic component of antioxidant protection (superoxide dismutase (SOD), catalase (CAT), glutathione peroxidase (GPx)) of saliva was studied following the generally accepted methods [1,2,18]. SOD activity was determined by accumulation of the epinephrine autoxidation product by O2-radical at pH > 7, the level of GPx was determined by the rate of glutathione consumption for the recovery of tert-Butyl hydroxide, and CAT activity was determined as per M.A. Korolyuk method [32].

### 2.10. Statistical Analysis

Statistical analysis of research results using the statistical research package for Windows 8.0. When comparing the values of the studied indicator in different groups, the Spearman rank correlation method was used. Comparison of empirical distributions of the trait was carried out using χ^2^—Pearson’s criterion. Evaluations between two independent choices for the level of quantitatively measured features were made using the nonparametric Mann–Whitney test. Significance of data differences for normal distribution series of analysis using Student’s *t*-test. All data were considered statistically significant at *p* < 0.05.

## 3. Results

### 3.1. Characteristics of the Chronic Generalized Periodontal Diseases and Healthy Groups

The study sample was composed of 40 females and 40 males. The clinical parameters confirmed a significant difference between the CGP and periodontally healthy groups (Table 1). Significant different scores were observed for BOP, OHI-S, and PD, CAL (*p* > 0.05). BOP index in patients with CGP ranged within 48.63 ± 3.51%, indicating increased vascular permeability, inflammation, alterative changes in gingival tissue, and the generalized presence of both spontaneous and initiated bleeding (>30% of the sites).

Monitoring of oral hygiene index (OHI-S) indicated a level of oral hygiene classified as ‘very poor’ (3.62 ± 0.46) in the CGP group, while the index in the healthy group was 1.32 ± 0.12.

### 3.2. Diameter and Blood Flow Rate Analysis of Gingival Microcirculation

**Figure 1:**
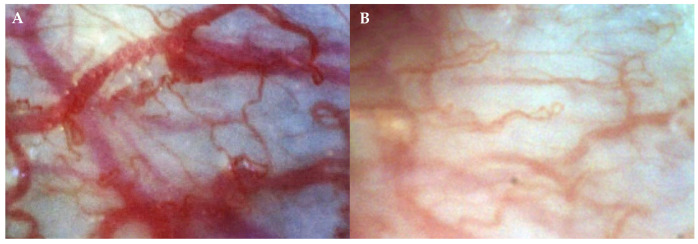
Capillaroscopic gingival microcirculation pictures of chronic generalized periodontitis subject (**A**) and healthy subject (**B**) (original magnification × 300).

The data were obtained analyzing capillaroscopic gingival pictures of chronic generalized periodontitis subjects and healthy subjects (Figure 1).

In the CGP group, the mean values of diameter of capillaries of arterial and transitional sections were 7.12 µm (standard deviation (SD) = 0.23) and 14.37 µm (SD = 0.56), respectively, versus 5.82 µm (SD = 0.16) and 8.65 µm (SD = 0.27) in the healthy group (Table 2). The opposite was observed in terms of the venous section of the capillaries. The proband indices in the CGP group were within 6.74 µm ± 0.52, which is significantly less than those of patients with healthy periodontium (11.43 µm ± 0.62).

There was a significant deviation from the results of healthy individuals, resulting in a significant decrease in the rate of linear blood flow in the CGP group. For example, the average value of the indicator in individuals with CGP in the arterial section was 347.72 ± 12.78 µm/s, with 309.82 ± 10.94 µm/s in the venous section, which was almost twice less than similar values of the healthy group (702.83 ± 14.75 µm/s and 635.56 ± 9.35 µm/s, respectively). During the analysis of indices of volume blood flow rate in the CGP subjects, a statistically significant decrease of the analyzed parameters was noted in arterial and venous sections (9824.73 ± 83.54 µm^3^/s and 23,763.73 ± 64.15 µm^3^/s) compared to the healthy subject data (50,738.74 ± 92.46 µm^3^/s for the arterial section and 54,863.21 ± 112.48 µm^3^/s for the venous section).

### 3.3. Vascular Endothelial Growth Factor analysis

Following the determination of Vascular Endothelial Growth Factor (VEGF) level in saliva, the results obtained in the CGP group were within the high values and averaged 1283.64 ± 58.32 mg/mL at 384.47 ± 21.96 mg/mL in the healthy group (Table 3).

### 3.4. Enzymatic Component Analysis

During the assessment of the condition of the enzymatic component of the saliva antioxidant protection, it was found that the SOD activity indices were significantly lower than the corresponding parameters in the healthy group, and roughly amounted to 54.39 ± 8.24 r.u. at 87.30 ± 12.9 r.u. in healthy patients (Table 4). The similar tendency is observed with respect to the content of CAT in the saliva. Patients with CGP demonstrated indices of 1.34 ± 0.45 MAT/L, while the intact patients had indices of 2.78 ± 0.64 MAT/L. In the vast majority of cases, the activity of GPx in the CGP subjects, was 1.5 times lower than the corresponding values of the healthy group and amounted to 24.73 ± 2.16 µmol/(min*L) (38.46 ± 2.74 µmol/(min*L) in the healthy group).

### 3.5. Cytokine Analysis

The analysis of cytokine profile of CGP patients’ blood serum demonstrated a significant increase of IL-1β, IL-6, TNF-α, IL-4 levels, as well as statistically significant decrease of IL-1α, IL-10 concentration (Table 5).

### 3.6. Immunoglobulin Analysis

A significant decrease in the content of sIgA in the saliva was observed in the patients of the CGP group in comparison with the healthy group (Table 6). In the vast majority of cases, patients with CGP demonstrated a dominant superiority of IgM and IgG levels, which are twice as high as at the healthy group (Table 6).

## 4. Discussion

**Figure 2:**
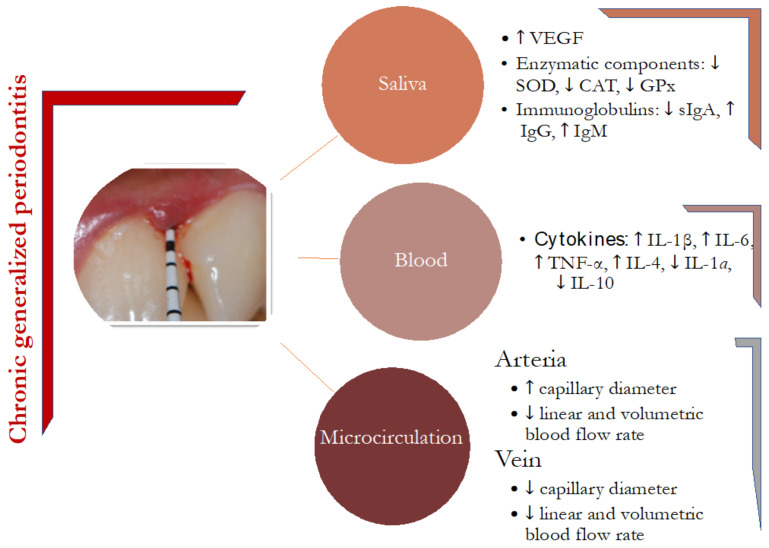
Characterization of observed changes in microcirculation, blood and saliva in chronic generalized periodontitis subject. GPx: glutathione peroxidase; Ig: immunoglobulin; IL: interleukin; CAT: catalase; SOD: superoxide dismutase; TNF: tumor necrosis factor; VEGF: vascular endothelial growth factor; ↑: increase; ↓: decrease.

The aim of this comparative study was to characterize the microcirculation, the expression of pro- and anti-inflammatory cytokines in periodontal tissues of CGP subjects and healthy periodontal subjects (Figure 2). Modern non-invasive diagnostic techniques were used, which allow an objective and complex assessment of hemodynamic processes not only at the level of capillaries themselves, but also at the level of arterioles and venules.

The increase of precapillary arterioles lumen concurrently with deformation and contraction of postcapillary venules diameter indicates the blood retention in tissues due to the outflow disorder, which is natural in the presence of persistent hypoxia and accumulation of inflammation mediators in the lesion.

The revealed microcirculatory dysfunctions in patients with CGP clearly demonstrate the progressive disorder of periodontal tissue perfusion and oxygenation, the presence of increased vascular permeability and functional failure of the microvascular system in the lesion. There was a decrease in perfusion of periodontal tissues and the development of obstructed outflow in the venous section of the microvascular system. The revealed changes indicate the functional inferiority of the periodontal vasculature and the presence of compensatory reactions for stabilization thereof. An ordered and linear blood flow was observed throughout the analyzed vessels. Concentric layers of fluid with different velocities moved smoothly against each other, forming a continuous laminar flow. In some cases, there were non-functioning capillaries with reduced lumen that had only plasma circulating. A pronounced vascularization of the transitory fold was determined in patients with CGP. In addition to the main components of microvasculature, a significant number of arteriovenous anastomoses was observed in which the blood flow was disordered and pendulous. In the absolute majority of cases, damaged capillaries with clear irregular edges and elements of perivascular edema were found in the CGP group, thus indicating vascular permeability disorder and the discharge of the liquid blood fraction outside the vessel into the interstitium. The revealed structural changes indicate a disorder of tissue blood filling and the transcapillary exchange inhibition.

A high level of VEGF in the saliva is regarded as a compensatory-adaptive mechanism that initiates and stimulates angiogenesis [33]. This fact confirms the conclusions on the inferiority of microcirculation in periodontal tissues in patients with CGP [34]. Significant index changes in patients with CGP are considered to be a compensatory and adaptive mechanism that has emerged in response to tissue perfusion disorder and is aimed at stimulation of neo-angiogenesis processes. The revealed disorders are indicated by significant diagnostic and prognostic criteria, which testify to the endothelium reactivity dysfunction, vascular insufficiency in the lesion, and the presence of persistent hypoxia [35,36].

Antioxidants are compounds that prevent the initiation or progression of oxidation reactions by trapping oxygen in the environment. Antioxidants are classified based on their mode of action as preventive antioxidants: e.g., SOD, CAT, GPx, glutathione reductase (GR), DNA repair enzymes [1]. Reactive oxygen species (ROS) form as a part of the physiological functions of all cells, and the significance of their role as mediators in cell signaling has become more evident [37]. Excessive ROS production plays a role in the pathogenesis of various chronic inflammatory diseases [38,39], including periodontal disease [40]. Cells and tissues require antioxidants to prevent the tissue damage caused by overproduction of ROS [41]. Analysis of the level of SOD, CAT, and GPx in the saliva indicates a decrease in the activity of the enzymatic component of the local antioxidant potential, which allows these parameters to be used as predictors of the escalation of the inflammatory process in periodontal disease [1,42,43]. The pronounced changes in the state of the enzymatic component of antioxidant protection allow to rigidly assume the activation of lipid peroxidation processes, formation of free oxygen radicals, disorganization of cell membranes and accumulation of tissue peroxidation by-products [18].

Both innate and adaptive immunity components are involved in the pathological process of CGP. Focal reaction to a bacterial infection activates innate immunity, producing a large number of cytokines and inflammatory mediators, which lead to the destruction of connective tissue and alveolar bone of the jaw [44]. Along with this, inhibition of local nonspecific protective factors is observed. Pro-inflammatory mediators, including cytokines, chemokines and metalloproteinases are known to increase dramatically in periodontal tissues and GCF in periodontitis [45,46]. Cytokines are produced by resident cells, such as epithelial cells and fibroblasts, and by phagocytes (neutrophils and macrophages) in the acute and early chronic phases of inflammation, and by immune cells (lymphocytes) in established and advanced lesions [47].

The study of cytokine profile condition in patients with CGP revealed a number of significant changes. Cytokine regulation of inflammation processes and immune response is necessary for development of appropriate protective reactions on pathogen permeation [48,49]. Dynamic equilibrium disorder in production of pro- and anti-inflammatory cytokines, their insufficient or excessive synthesis leads to pathological process development [50,51].

Serum cytokines can come from a variety of cells; it may be leakage from the microcirculation or endothelial activation from the release of microbial factors from the oral cavity. Indeed, periodontopathogens can migrate and invade the epithelium and then the connective tissue to finally reach the bloodstream [52,53]. The bacterial products (exotoxins and endotoxins) can also reach the blood circulation and, thus, exert their toxicity remotely. Bacteria or bacterial products thus triggers an immune response. The pro-inflammatory molecules produced are therefore found in the systemic circulation and are able to diffuse and cause other pathologies such as cardiovascular diseases, diabetes, rheumatoid arthritis, cancer [53,54]. The pronounced increase in IL-1β production in patients with CGP is probably caused by massive stimulation of cells of monocyte–macrophage system by antigens of pathogenic flora and indicates the development of purulent infection [55]. The content of IL-6, a mediator of the acute phase causing Cluster of Differentiation 19+ (CD19+) activation, proved to be significantly higher than the corresponding parameter of the healthy group. The intensity of IL-6 production in the examined patients is characterized by the intensity of adaptive immunity functioning. Significant excess of cytokine IL-4 in the blood serum of the CGP group is considered as a compensatory anti-inflammatory reaction aimed at limiting hyperreaction of immunocompetent cells [56]. At the same time, IL-4 stimulates CD4+ to differentiate towards Th2, activate CD19+ and convert them to plasmocytes, as well as to intensify the processes of antibody synthesis against foreign invaders. The CGP Group showed a significant decrease in the level of IL-10, a powerful anti-inflammatory and immunosuppressive factor that inhibits the synthesis of pro-inflammatory cytokines by activated macrophages and Th1. This decline indicates a manifestation of inflammatory processes and inhibition of mechanisms to limit and eliminate it. Moreover, IL-10 has a key function in the osteoclastic bone resorption and the regulation of osteoblastic bone formation [57]. Taiete et al. (2019) demonstrated that in generalized aggressive periodontitis patients treated with scaling and root planning, a higher IL-10 concentration at baseline was associated with a higher reduction in probing pocket depth at 6 months [58]. One recent study has demonstrated, in an animal model, which IL-10 can modulate local host immune responses and prevent inflammatory damage of alveolar bone by reducing pro-inflammatory cytokine expression and decreasing local proliferation of Th17 cells [59]. Thus, IL-10 could be a therapeutic strategy in periodontitis [57]. Statistically significant decrease in the content of IL-1ra, a cytokine that competes with the pro-inflammatory cytokine IL-1β for the receptor on immunocompetent cells and prevents its binding to it, was revealed in patients with CGP. Significant increase in the pro-inflammatory cytokine TNF-α, which potentiates inflammatory processes and is capable of affecting periodontal tissues in isolation, causing alteration, was observed in the patients of the CGP group. Regarding TNF-α, there is an evidence of possible participation in the inflammatory resorption of the alveolar bone by affecting the surface receptors of osteoclasts and stimulating them to resorb calcium ions [60,61].

The study of humoral immunity markers has demonstrated an increase in inflammatory processes in damaged periodontal tissues, as well as the development of reactions to eliminate and limit them. High level of IgM and IgG indicates the presence of an acute inflammatory reaction and the intensity of the current CGP recurrence. As a result of the analysis of local oral immunity condition, data demonstrated significant shifts in the humoral component of nonspecific and specific immunity. Thus, a significant decrease in the content of sIgA in the saliva was observed in the patients of the CGP group in comparison with the healthy group. This indicates a decrease in local mucosal immunity and may be associated with an imbalance between its production and catabolism, as well as transport disorder through the mucous membrane epithelium [62]. Generally, patients with CGP demonstrated higher expression of IgM and IgG levels, which were twice as high as at the healthy group. This indicates the presence of an acute inflammatory process, the intensity of the response and the activation of antibody-dependent cytolysis processes. Numerous researches show that IgG and IgM synthesized in response to microbial contamination can cross-react with antigens of damaged periodontal tissues, becoming one of the components in the vicious cycle of immune-mediated inflammation [24,63].

The sIgA level in the saliva of the patients of the CGP group was significantly lower than the compared indicators of the control group. The results for sIgA seems surprising then does not include results reported by other studies [64,65]. Discrepancies between these results could be attributed to differences in study designs and sampling—tobacco [64] and diabetic [65] subjects—and measuring techniques. Our assessment was precisely quantitative, the results are reliable and fit into the standards proposed by the manufacturer. Deficiency of sIgA indicates an inferiority of the local resistive potential, and is probably due to functional insufficiency of plasma cells of the mucous membranes, impaired translocation of serum IgA through the epithelium and its binding to the secretory component and protective mucins. A similar decrease in sIgA levels in patients with inflammation is natural and triggered by exacerbation of periodontitis, as well as cytokine dissonance, pro-inflammatory cytokines are known to inhibit the production of sIgA.

Although the main results of this study are based only on the comparison between the CGP and healthy patients, strength of this innovative research resides in using computer capillaroscopy, which is one of the auxiliary diagnostic methods of research that allows to observe the functioning of the peripheral part of the human cardiovascular system in the skin and mucous membranes. Computer processing provides the following advantages: visualization of microcirculatory changes in capillary blood flow on the monitor screen; enhancement of image contrast; measurement of the size of capillaries, the number of blood cells aggregates; observation of capillary blood flow in dynamics. Applied to microcirculation periodontal environment, computer capillaroscopy made it possible to establish the nature of the disturbances in the microvasculature in patients with CGP and makes it possible to switch from a visual assessment of capillary blood flow to obtaining its numerical characteristics, in particular, the diameter of capillaries and the velocity of capillary blood flow. This method allows non-invasive assessment of the angioarchitectonics of the capillary bed (the shape of the capillaries, the density of the capillary network and the area of the oxygen-exchange surface) at low magnifications, as well as the size of single capillaries (the diameter of the arterial, transitional and venous sections) and hemodynamic characteristics of the blood flow (linear and volumetric velocities) in isolation in the arterial and venous sections. At least, all processes, in our study, were carried out for two researchers, who were blinded to the participants’ periodontal status.

The novelty of our study lies in the evaluation inflammatory markers that have not been previously analyzed in microvascular lesions of CGP. However, a limitation of the present study was that age range of the study population is restricted and merited, as for gender, complementary analysis based on an adjusted model. Aging is a known process that increases oxidative stress and induces low-grade inflammation that influences micro-circulation. Gender should have been considered. Moreover, even if no significant correlation between caries and periodontitis has been observed in the previous studies, the two conditions have a common etiology [66,67]. Thus, the influence of caries in the current results could also have been considered, as well as the oral hygiene status and the influence of tooth loss. This could independently cause a disruption of the microcirculation and an unhealthy condition of the oral cavity. In this case, inflammation could influence both microcirculation and periodontitis, even though there is no association between micro-circulation disruption and health status. Secondly, a possible bias, caused by refractive errors and variations in vessel diameter throughout the cardiac cycle should be considered. To minimize the risk, patients have been carefully selected. The manifestation of diseases of the cardiovascular system in patients was excluded. Initially, an examination was carried out that is part of the compulsory health insurance: electrocardiography, echocardiography, ultrasound examination of the vessels of the head and neck, clinical and biochemical blood tests, analysis for glycated hemoglobin, coagulogram and lipid profile, analysis of cholesterol levels, triacylglycerides, high- and low-density lipoproteins, C- reactive protein, rheumatoid factor.

## 5. Conclusions

The modifications observed on adults presenting periodontal diseases indicate the functional inferiority of the periodontal vascular system and the presence of compensatory reactions for its stabilization. These results are consistent with the body of evidence suggesting a correlation between microvascular changes and local and systemic markers of inflammation. The study of the condition of periodontal microcirculation offers new perspectives to understand the role of vascular disorders in the pathogenesis of CGP. Applications for future research could consist in applying similar protocols to people at high risk of periodontal diseases, such as diabetic subjects or subjects with systemic diseases.

## Figures and Tables

**Table 1:** Baseline Clinical Features of the Subjects.

Index	CGP Group (*n* = 40)	Healthy Group (*n* = 40)
Age ^a^	49.2 ± 4.3	49.7 ± 4.8
Missing teeth BOP ^a^ (%)	4.9 ± 3.2 48.63 ± 3.51 **	4.3 ± 2.4 5.24 ± 0.37
OHI-S ^a^	3.62 ± 0.46 *	1.32 ± 0.12
PD ^a^ (mm)	4.25 ± 0.73 *	0.8 ± 0.23
CAL ^a^ (mm)	3.27 ± 0.04 *	0.4 ± 0.01

The values are means ± standard deviations. BOP: bleeding on probing; OHI-S: oral hygiene index-simplified; CAL: clinical attachment level; CGP: chronic generalized periodontitis; PD: pocket depth. ^a^ The Student’s *t*-test was used to compared the CGP and the healthy groups; * *p* < 0.01; ** *p* < 0.001.

**Table 2:** Microcirculation indices in the transitory fold of the oral cavity in patients with chronic generalized periodontitis.

Index	CGP Group (*n* = 40)	Healthy Group (*n* = 40)
Capillary diameter in the arterial section ^a^ (µm)	7.12 ± 0.23	5.82 ± 0.16
Capillary diameter in the interjacent section ^a^ (µm)	14.37 ± 0.56 *	8.65 ± 0.27
Capillary diameter in the venous section ^a^ (µm)	6.74 ± 0.52 *	11.43 ± 0.62
Linear blood flow rate in the arterial section ^a^ (µm/s)	347.72 ± 12.78 **	702.83 ± 14.75
Linear blood flow rate in the venous section ^a^ (µm/s)	309.82 ± 10.94 **	635.56 ± 9.35
Volumetric blood flow rate in the arterial section ^a^ (µm^3^/s)	9824.73 ± 83.54 **	50,738.74 ± 92.46
Volumetric blood flow rate in the venous section ^a^ (µm^3^/s)	23,763.73 ± 64.15 **	54,863.21 ± 112.48

^a^ The nonparametric Mann–Whitney test and Spearman rank correlation method were used; * *p* < 0.05; ** *p* < 0.01. CGP: chronic generalized periodontitis.

**Table 3:** Level of vascular endothelial growth factor (VEGF) in saliva in patients with chronic generalized periodontitis.

Index	CGP Group (*n* = 40)	Healthy Group (*n* = 40)
VEGF ^a^ (mg/mL)	1283.64 ± 58.32 ***	384.47 ± 21.96

^a^ The nonparametric Mann–Whitney test and Spearman rank correlation method were used; *** *p* < 0.001.

**Table 4:** Activity indices of the enzymatic component of the saliva antioxidant protection in patients with chronic generalized periodontitis. CGP: chronic generalized periodontitis.

Enzyme	CGP Group (*n* = 40)	Healthy Group (*n* = 40)
SOD ^a^ (r.u.)	54.39 ± 8.24 **	87.30 ± 12.92
CAT ^a^ (МАТ/L)	1.34 ± 0.45 *	2.97 ± 0.64
GPx ^a^ (µmol/(min*L))	24.73 ± 2.16 *	38.46 ± 2.74

^a^ The nonparametric Mann–Whitney test and Spearman rank correlation method were used; * *p* < 0.05; ** *p* < 0.01.

**Table 5:** Levels of anti-inflammatory cytokines in serum in patients with chronic generalized periodontitis.

Anti-Inflammatory Cytokine	CGP Group (*n* = 40)	Healthy Group (*n* = 40)
IL-1β ^a^ (pg/mL)	19.7 ± 3.2 **	3.6 ± 1.01
IL-6 ^a^ (pg/mL)	73.29 ± 5.11 **	4.52 ± 0.81
TNF-α ^a^ (pg/mL)	13.68 ± 2.39 **	1.24 ± 0.22
IL-4 ^a^ (pg/mL)	0.154 ± 0.021 **	0.034± 0.007
IL-10 ^a^ (pg/mL)	2.34 ± 0.92 *	7.02 ± 1.26
IL-1α ^a^ (pg/mL)	248.56 ± 4.31 **	436.72± 6.28

^a^ The nonparametric Mann–Whitney test and Spearman rank correlation method were used; * *p* < 0.05; ** *p* < 0.01. CGP: chronic generalized periodontitis; IL: interleukin; TNF: tumor necrosis factor.

**Table 6:** Concentration of immunoglobulins in saliva in patients with chronic generalized periodontitis.

Immunoglobulin	CGP Group (*n* = 40)	Healthy Group (*n* = 40)
sIgA ^a^ (mkg/mL)	346.8 ± 33.7 **	912.5 ± 74.5
IgG ^a^ (mkg/mL)	66.47 ± 20.79 *	39.28 ± 16.92
IgM ^a^ (mkg/mL)	82.32 ± 21.24 **	43.81 ± 12.65

^a^ The nonparametric Mann–Whitney test and χ^2^—Pearson’s criteria were used; * *p* < 0.05; ** *p* < 0.01. CGP: chronic generalized periodontitis; Ig: immunoglobulin.

## Data Availability

The data presented in this study are available on request from the corresponding author.

## Supplementary Materials

The following are available online at https://www.mdpi.com/2077-0383/10/6/1262/s1, Figure S1: Capillaroscopy. (A) Capillaroscope KK4-01 connected to a computer. (B) Capillaroscopy of gingival tissue using the capillaroscope KK4-01.
