# Supplementary material for: Evaluation of Microcirculation, Cytokine Profile, and Local Antioxidant Protection Indices in Periodontal Health, and Stage II, Stage III Periodontitis
Source: J Clin Med. 2021 Mar 18;10(6):1262. doi: 10.3390/jcm10061262 (PMC8003283; doi:10.3390/jcm10061262)

Evaluation of microcirculation, cytokine profile and local antioxidant protection indices in periodontal health and chronic generalized periodontitis

Artem Eldzharov, Dzerassa Kabaloeva, Dmitry Nemeryuk, Aida Goncharenko, Adelina Gatsalova, Elena Ivanova, Igor Kostritskiy, Florence Carrouel and Denis Bourgeois

Supplementary Figure 1. Capillaroscopy. A. Capillaroscope KK4-01 connected to a computer. B. Capillaroscopy of gingival tissue using the capillaroscope KK4-01.

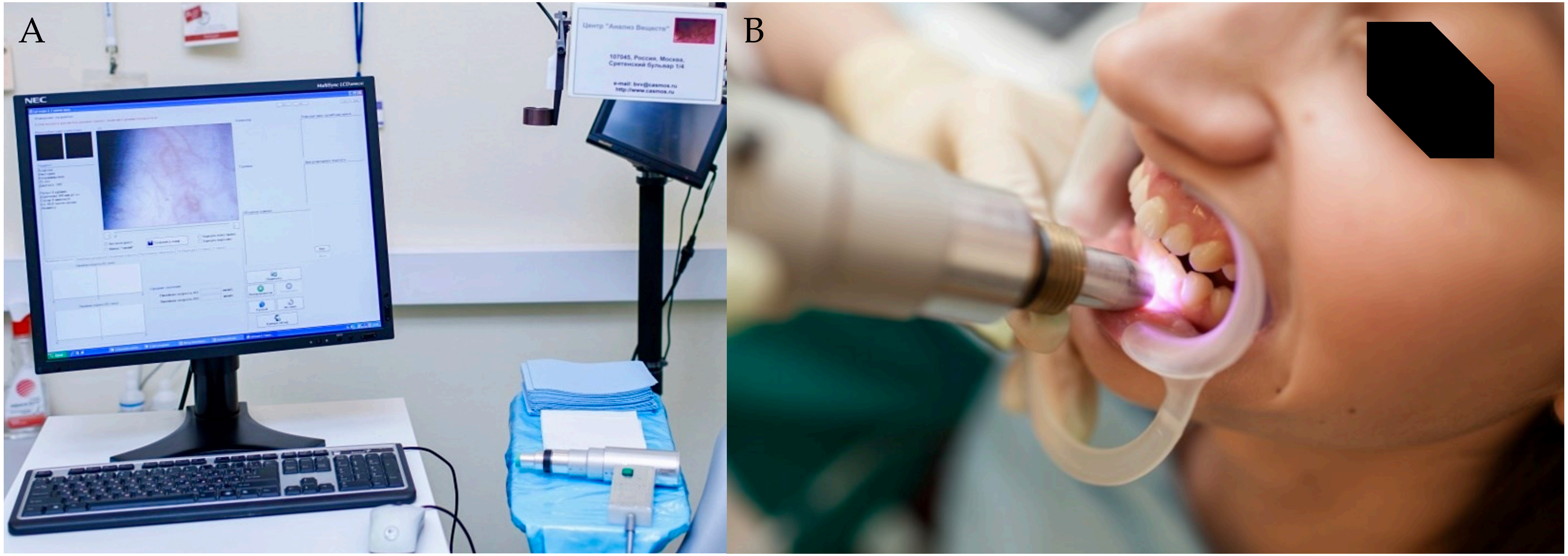

Supplement: Supplementary file 1 [file jcm-10-01262-s001.pdf]
